# Etoposide-induced DNA damage affects multiple cellular pathways in addition to DNA damage response

**DOI:** 10.18632/oncotarget.24517

**Published:** 2018-02-16

**Authors:** Fengxiang Wei, Peng Hao, Xiangzhong Zhang, Haiyan Hu, Dan Jiang, Aihua Yin, Lijuan Wen, Lihong Zheng, Jeffrey Zheru He, Wenjuan Mei, Hui Zeng, Damu Tang

**Affiliations:** ^1^The Genetics Laboratory, Shenzhen Longgang District Maternity and Child Healthcare Hospital, Shenzhen, Guangdong, PR China; ^2^ Division of Nephrology, The First Affiliated Hospital of Jiamusi University, Jiamusi, Heilongjiang, PR China; ^3^ Department of Hematology, The Third Affiliated Hospital of Sun Yat-Sen University, Guangzhou, Guangdong, PR China; ^4^ Department of Obstetrics and Gynecology, Shenzhen Maternal and Child Healthcare Hospital, Southern Medical University, Shenzhen, Guangdong, PR China; ^5^ Shenzhen Hua Da Clinical Laboratory Center Co., Ltd., Shenzhen, Guangdong, PR China; ^6^ Maternal and Children Metabolic-Genetic Key Laboratory, Guangdong Women and Children Hospital, Guangzhou, PR China; ^7^ Zunyi Medical University, Zunyi, Guizhou, PR China; ^8^ Department of Biogenetics, Qiqihar Medical University, Qiqihar, Heilongjiang, PR China; ^9^ Harvard Medical School and Massachusetts General Hospital, Boston, MA, USA; ^10^ Department of Nephrology, The First Affiliated Hospital of Nanchang University, Nanchang, Jiangxi, PR China; ^11^ Department of Thoracic Surgery, Fourth Hospital of Hebei Medical University, Shijiazhuang City, Hebei, PR China; ^12^ Division of Nephrology, Department of Medicine, McMaster University, Hamilton, ON, Canada

**Keywords:** etoposide, DNA damage response, gene expression, RNA sequencing

## Abstract

DNA damage response (DDR) coordinates lesion repair and checkpoint activation. DDR is intimately connected with transcription. However, the relationship between DDR and transcription has not been clearly established. We report here RNA-sequencing analyses of MCF7 cells containing double-strand breaks induced by etoposide. While etoposide does not apparently cause global changes in mRNA abundance, it altered some gene expression. At the setting of fold alteration ≥ 2 and false discovery rate (FDR) ≤ 0.001, FDR < 0.05, or *p* < 0.05, etoposide upregulated 96, 268, or 860 genes and downregulated 41, 133, or 503 genes in MCF7 cells. Among these differentially expressed genes (DEGs), the processes of biogenesis, metabolism, cell motility, signal transduction, and others were affected; the pathways of Ras GTPase activity, RNA binding, cytokine-mediated signaling, kinase regulatory activity, protein binding, and translation were upregulated, and those pathways related to coated vesicle, calmodulin binding, and microtubule-based movement were downregulated. We further identified RABL6, RFTN2, FAS-AS1, and TCEB3CL as new DDR-affected genes in MCF7 and T47D cells. By metabolic labelling using 4-thiouridine, we observed dynamic alterations in the transcription of these genes in etoposide-treated MCF7 and T47D cells. During 0-2 hour etoposide treatment, RABL6 transcription was robustly increased at 0.5 and 1 hour in MCF7 cells and at 2 hours in T47D cells, while FAS-AS1 transcription was dramatically and steadily elevated in both cell lines. Taken together, we demonstrate dynamic alterations in transcription and that these changes affect multiple cellular processes in etoposide-induced DDR.

## INTRODUCTION

DNA damage response (DDR) is the mechanism that guards genome integrity and ensures the faithful transmission of the genetic codes to the next generation cells [1]. Lying in the center of DDR are three PI3 kinase-related kinases (PIKKs), ATM, ATR, and DNA-PK [2]. ATR is typically activated by single strand DNA (ssDNA) lesions, and is required for maintaining genome stability [3, 4]; both ATM and DNA-PK are activated by double strand DNA breaks (DSBs), and play essential roles in DSB repair [1, 5, 6]. All three PIKKs, particularly ATM and ATR, preserve genome integrity through coordination of checkpoint activation and DNA lesion repair.

While checkpoint activation and DNA lesion repair are the core components of DDR, repair of DNA lesions or maintenance of genome stability is clearly much more pervasive, in which multiple cellular processes are involved [7]. Cell metabolism is intimately connected with DDR [8, 9]; intra- and inter-cellular communications are taking place to pass the message of DDR within cells and in their surrounding [10]; and in the same time preparation is under way for cells to re-enter cell cycle upon lesions being repaired. Collectively, the execution of DDR requires a much broader coordination. This concept is in accordance with the knowledge that DNA damage is induced by multiple sources including external genotoxic materials, internal metabolic products, DNA replication, and RNA metabolism.

Differential regulation of mRNA translation and RNA processing is a major feature of DDR. Both UV and ionizing radiation (IR) selectively inhibit and enhance a set of protein translation through excluding and recruiting mRNA species to polysomes [11, 12]. This selection facilitates the translation of proteins involved in DNA repair [12] and also affects multiple cellular processes [11, 13]. Likewise, selective modulation of gene expression was also demonstrated in other aspects of RNA metabolism, including RNA slicing and processing via the involvement of a set of specific RNA processing factors [14, 15], polyadenylation [16, 17], and export [18, 19]. The collective effects of these selective regulations of RNA metabolism are to ensure the expression of genes involved in DNA damage repair while inhibition of others [19].

The above theme remains with respect to gene transcription. While there is evidence suggesting a global transcription inhibition under DNA damage [20, 21], it is well-established that transcription of p53 targets is upregulated, including p21^CIP1^ (CDKN1A), BAX, MDM2, and PUMA [22, 23]. Despite this knowledge, our understanding of the alterations in gene expression during DDR remains unclear. There are also reports favoring local rather than global repression of gene transcription in response to DNA damages [24–27]. Additionally, profiling of gene expression in U87 cells treated with IR revealed a correlation coefficient of 0.92 in mRNA abundance between radiated and non-radiated cells, an observation that is not in line with global transcription repression [28]; IR-upregulated genes were detected both *in vitro*, *in vivo* (xenograft tumors), and independent of the p53 status [28–30]; and an elevation in IR dose decreased the level of transcription upregulation [30].

Although modulation of gene expression is an important aspect of DDR, this aspect has not been thoroughly investigated. To advance our understanding on this process, we have profiled gene expression of MCF7 cells treated with etoposide (ETOP) using the state-of-the-art RNA sequencing technology and performed a thorough pathway analysis on differentially expressed genes (DEGs). We report here that ETOP-induced double-strand breaks (DSBs) affect gene expression in multiple cellular pathways. During this effort, five novel DDR-affected genes were identified and their transcription kinetics in MCF7 and T47D cells treated with ETOP were investigated.

## RESULTS

### ETOP-induced DNA damage does not associate with global alterations in RNA abundance

**Figure 1:**
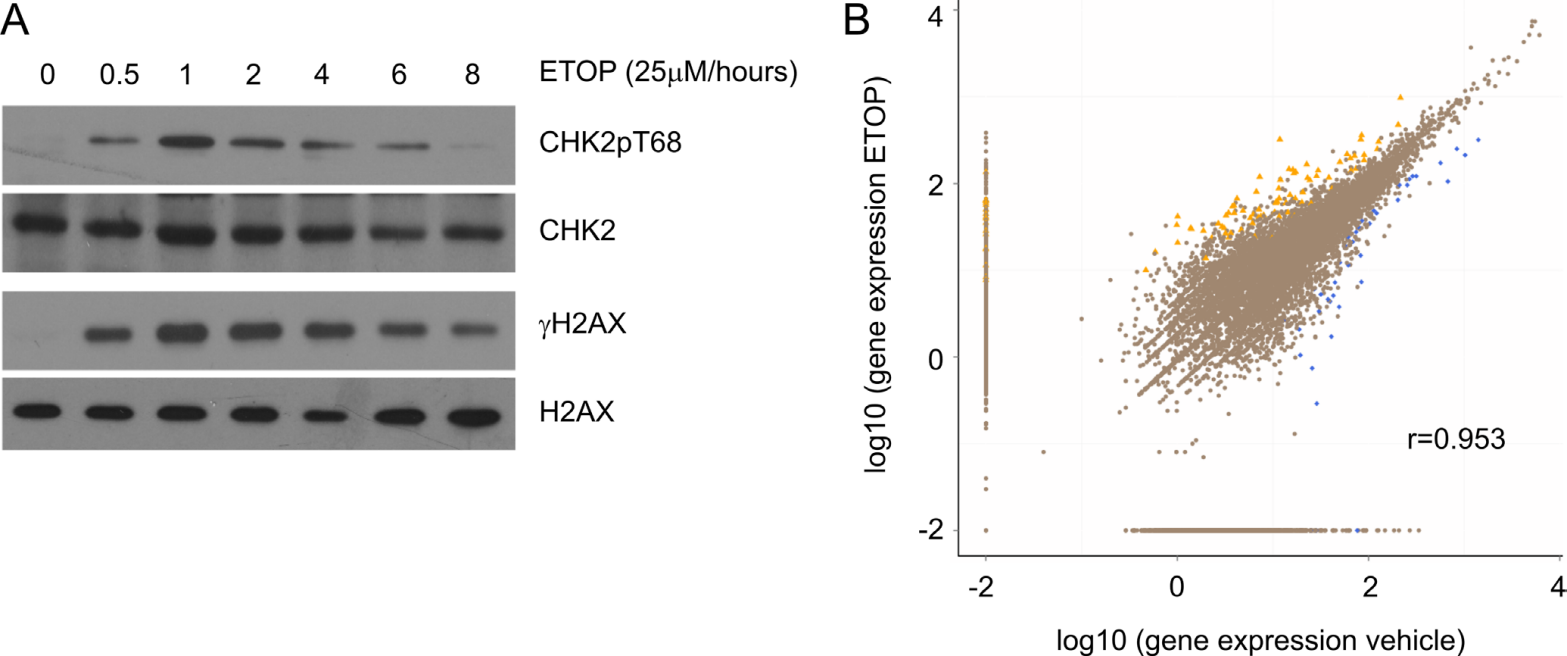
Global gene expression under ETOP-induced DNA damage (**A**) MCF7 cells were treated with either vehicle (DMSO or 0) or ETOP (25μM) for the indicated period of time, followed by Western blot examination for CHK2 phosphorylation at threonine 68 (CHK2pT68), CHK2, γH2AX, and H2AX. (**B**) MCF7 cells were treated with vehicle or ETOP (25μM) for 2 hours, followed by RNA sequencing analysis. Transcripts were quantified as FPKM. The number of transcript pairs (vehicle vs ETOP) was 12,324. The correlation coefficient (r) was indicated. Differentially expressed genes (DEGs) were defined as log2 ratio of ETOP/vehicle ≥ 1 and FDR ≤ 0.001. The number of upregulated transcripts (yellow triangles) is *n* = 96; the number of downregulated DEGs (blue squares) is *n* = 41.

Recent developments clearly reveal an intimate connection between RNA metabolism and DDR [19, 31–33]. Transcription is a major factor regulating transcript abundance, and is associated with DNA damage. To counter transcription-produced DNA damage, a repair program, transcription-coupled repair (TCR), was developed in cells [34, 35]. Interestingly, DSBs induced by topoisomerase IIβ have been shown to be an essential component in estrogen-initiated transcription [36] and ETOP-induced DSBs enhance AIRE (autoimmune regulator)-mediated transcription [37]. Although transcript abundance in cells containing IR-induced DSBs has been profiled by cDNA microarray [28–30], it remains unclear how gene expression is associated with DDR. This issue is particularly relevant considering the general believe of global repression of transcription by DNA damage [24–27]. To further examine this concept, we first determined the kinetics of ETOP-induced DDR in MCF7 cells. ETOP is a well-established topoisomerase inhibitor and induces DSBs [38, 39]. At 2 hours (h), ETOP clearly induced DSBs evidenced by the appearance of γH2AX (Figure 1A) and based on comet assay reported in our previous publication [40]. An increase in CHK2 phosphorylation at threonine 68 further confirmed DDR (Figure 1A). We thus performed an RNA sequencing analysis of MCF7 cells treated with vehicle or ETOP for 2 hours. Scatter plot analysis of 12,324 pairs of transcripts using the normalized FPKM (fragments per kilobase of exon per million fragments mapped) demonstrated a high level of similarity between vehicle- and ETOP-treated cells with the correlation coefficient being 0.953 (Figure 1B). These observations suggest that global transcription is unlikely suppressed by ETOP-induced DNA damage. Our results confirmed a previous observation that cDNA microarray analysis revealed a correlation coefficient of 0.92 between mock-treated U87 cells and IR-stimulated cells with respect to mRNA abundance [28]. However, this concept does not exclude the possibility that global transcription is repressed under massive DNA damage.

### Alterations of multiple pathways in MCF7 cells treated with ETOP

**Figure 2:**
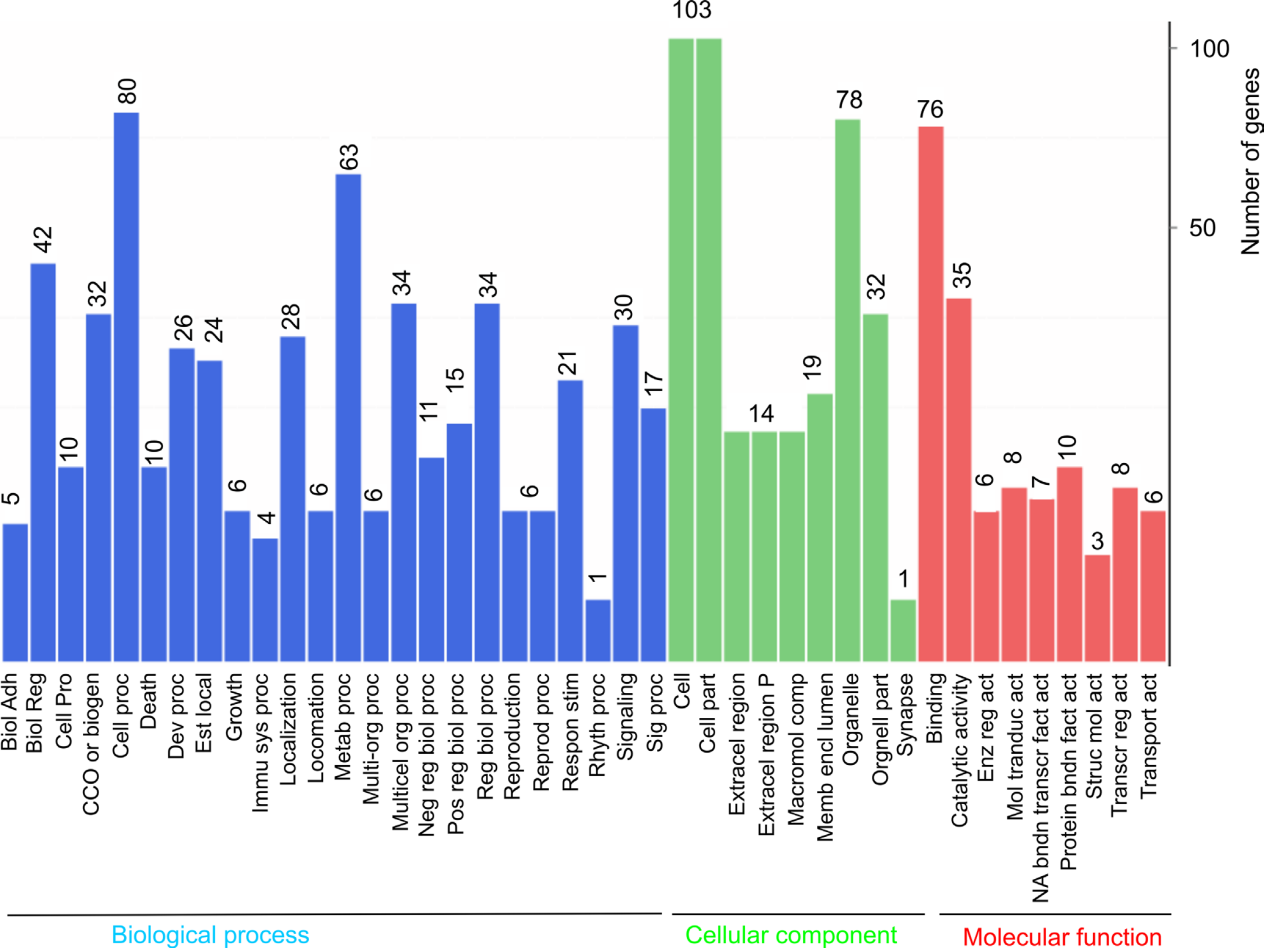
Gene ontology enrichment analysis of DEGs DEGs described in Figure 1 legend were analyzed for enrichment in three GO ontologies: biological process, cellular component, and molecular function. The number of genes enriched in individual GO terms is indicated on top of the individual bars. Biol Adh: biological adhesion; Biol reg: biological regulation; Cell pro: cell proliferation; CCO or biogen: cellular component organization or biogenesis; Cell proc: cellular process; Dev proc: developmental process; Est local: establishment of localization; Immu sys proc: immune system process; Metab proc: metabolic process; Multi-org proc: multi-organism process; Multicel org proc: multicellular organismal process; Neg reg biol proc: negative regulation of biological process; Pos reg biol proc: positive regulation of biological process; Reg biol proc: regulation of biological process; Reprod proc: reproductive process; Respon stim: response to stimulus; Rhyth proc: rhythmic process; Sig proc: signaling process; Extracel region: extracellular region; Extracel region P: extracellular region part; Macromol comp: macromolecular complex; Memb encl lumen: membrane-enclosed lumen; Enz reg act: enzymatic regulator activity; Mol transduct act: molecular transducer activity; NA bndn transcr fact act: nucleic acid binding transcription factor activity; Protein bndn fact act: protein binding factor activity; Struc mol act: structural molecule activity; Transcr reg act: transcription regulator activity; and Transport act: transporter activity.

Although our observations do not support a global repression of transcription in cells containing DSBs (Figure 1B), a small set of genes were up- and down-regulated in MCF7 cells treated with ETOP at the setting of log2 ratio of treatment/control ≥ 1 (2 fold alteration) and FDR ≤ 0.001 (Figure 1B). These differentially expressed genes (DEGs) include 96 upregulated genes and 41 downregulated genes in ETOP-treated MCF7 cells (Supplementary Table 1, Table 1) and these DEGs may contribute to a broader aspect of DDR than the classical components: checkpoint activation and lesion repair. To examine this possibility, we carried out a gene ontology (GO) enrichment analysis, which showed an enrichment in the three GO ontologies (molecular function, cellular component, and biological process) and multiple terms within individual GO ontology in MCF7 cells treated with ETOP (Figure 2). Evidence suggests that these three GO ontologies are affected in cells undergoing DDR. The biological processes of GO ontology, including cellular component organization (CCO or biogen), immunological process, and metabolic process [41–44], are regulated by DDR. For the cellular components of GO ontology, “cell” (including cell membrane) [45], “cell part” (cellular components) [46], and “organelle” (including the nucleus, mitochondria, cytoskeleton, and others) [47–49] are contributors to DDR. The molecular function of GO ontology includes 1) protein interactions (“binding”) which plays an essential role in the activation of ATM, ATR, and DNAPK [50, 51], and 2) “catalytic activity” in which a variety of posttranslational modifications are critical for DDR [52, 53]. Collectively, the above observations support a broader impact of DDR on multiple cellular functions and systems; their alterations in turn contribute to a variety of aspects of DDR. However, while the above GO analysis provides a general concept for the involvement of broad systems in ETOP-induced DDR, the analysis does not illustrate details on how these systems are engaged.

**Table 1:** Down-regulated genes in ETOP-treated MCF7 cells^1^

Gene	locus	Log2 Ratio	*P* value	FDR
TCEB3CL	18q21.1	–12.89	2.27E-09	4.31E-07
CTAGE15	7q35	–11.47	7.42E-06	0.000602
PLGLB1	2p11.2	–11.28	7.42E-06	0.000598
MFAP5	12p13.31	–6.63	1.70E-06	0.000193
TSPAN11	12p11.21	–5.12	1.04E-09	2.04E-07
MRPS31P5	13q14.3	–4.57	1.88E-07	2.63E-05
GRIN1	9q34.3	–4.20	9.32E-06	0.000718
CPLX1	4p16.3	–3.71	2.11E-06	0.00023
RIMS1	6q13	–3.52	3.92E-27	6.05E-24
CHN2	7p14.3	–3.18	5.66E-06	0.000502
SLC9A8	20q13.13	–3.18	5.66E-06	0.000499
TM4SF18	3q25.1	–3.17	3.01E-06	0.000304
PROB1	5q31.2	–3.07	5.82E-07	7.63E-05
LCORL	4p15.31	–3.03	1.08E-06	0.000132
MTRNR2L10	Xp11.21	–2.68	6.18E-28	1.27E-24
SUPT20HL1	Xp22.11	–2.63	1.26E-05	0.000927
LRRC8E	19p13.2	–2.59	1.26E-05	0.000933
TSEN54	17q25.1	–2.48	1.44E-06	0.000166
KGFLP2	9p12	–2.41	1.91E-10	4.37E-08
FMN1	15q13.3	–2.29	4.35E-06	0.0004
MTRNR2L8	11p15.4	–2.27	5.12E-39	2.10E-35
FGF2	4q28.1	–2.16	2.24E-06	0.000238
MTRNR2L2	5q14.1	–2.14	3.64E-40	2.25E-36
LRRC37A2	17q21.31	–2.03	3.76E-08	6.02E-06
MTRNR2L1	17p11.2	–1.74	8.59E-28	1.51E-24
PFN1P2	1p11.2	–1.72	4.60E-23	5.15E-20
DMTF1	7q21.12	–1.68	5.67E-06	0.000492
speedy hom E8, pseudo^2^	NA	–1.65	1.09E-12	3.94E-10
TOX4	14q11.2	–1.57	7.73E-10	1.59E-07
ZNF710	15q26.1	–1.56	7.26E-06	0.000593
DNAJC18	5q31.2	–1.43	3.40E-07	4.60E-05
RHOQ	2p21	–1.41	7.32E-10	1.53E-07
HAS3	16q22.1	–1.41	6.36E-06	0.000529
FLJ31306	#N/A	–1.38	4.80E-13	1.79E-10
ICMT	1p36.31	–1.35	1.35E-05	0.000976
TSHZ2	20q13.2	–1.31	4.37E-14	2.00E-11
LOC100133286	21q22.12	–1.26	9.27E-06	0.000718
URAHP	16q24.3	–1.25	5.95E-06	0.000502
EMILIN3	20q12	–1.23	1.02E-06	0.000125
TSR1	17p13.3	–1.19	2.62E-06	0.000269
SYNC	1p35.1	–1.14	2.97E-09	5.47E-07

1: The candidates were selected based on log2 ratio (ETOP/vehicle) ≤ −1 and FDR ≤ 0.001.

2: speedy homolog E8 (Xenopus laevis), pseudogene; unable to locate its locus (NA).

We thus examined the specific pathways affected by ETOP treatment. For this purpose, we have relaxed the stringency used to identify DEGs from FDR ≤ 0.001 to FDR < 0.05 or *p* < 0.05 while maintaining log2 ratio ≥ 1. At FDR < 0.05, the number of the upregulated genes increases to 268 (Supplementary Table 2), and the number of downregulated genes is up to 133 (Supplementary Table 3). Further relaxation to *p* < 0.05 resulted in increases in upregulated genes and downregulated genes to 860 and 503, respectively (Supplementary Tables 4, 5). The above relax conditions are justified. MCF7 cells express wild type p53, evidenced by p53 stabilization and the upregulation of a well-established p53 target p21^CIP1^ encoded by the CDKN1A gene in ETOP-treated MCF7 cells [39, 54]. As expected, upregulation of CDKN1A (log2 ratio = 2.5, *p* = 0.0068, and FDR = 0.106) is defined at *p* < 0.05 (Supplementary Table 4). MDM2 is also a p53 target induced by IR [22, 23, 55], and was upregulated (log2 ratio = 1.08, *p* = 0.0008, and FDR = 0.026) in ETOP-treated MCF7 cells under the relax condition of FDR < 0.05 (Supplementary Table 2).

**Figure 3:**
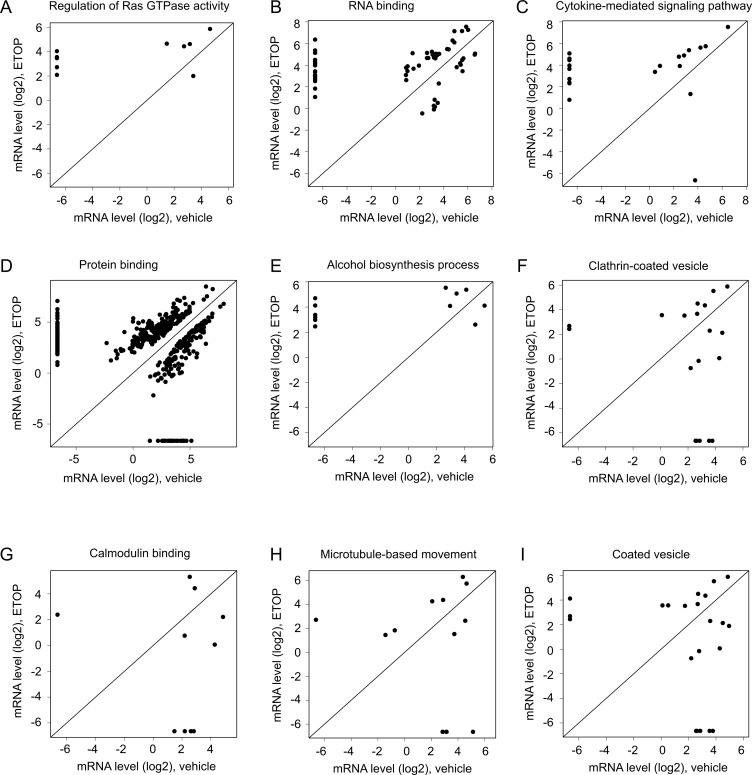
Alterations in gene expression in the enriched gene sets Enrichment analysis for those DEGs defined as *p* < 0.05 (Supplementary Table 4) was performed using the GAGE package in R [56]. Scatter plots show alterations in gene expression for individual genes in the upregulated gene sets (**A–E**) and downregulated gene sets (**F–I**). Gene expression was quantified as log2 units. See Supplementary Tables 6–18 for the individual gene sets.

With the largest set of DEGs defined (log2 ratio ≥ 1, *p* < 0.05; Supplementary Tables 4, 5), we performed gene set and pathway enrichment analyses using the GAGE and Reactome packages in R [56, 57]. The gene sets used in these analyses were derived from the GO term group (go.sets.hs). Analyses using the Gage package in R identified upregulations in ETOP-treated MCF7 cells in gene sets regulating Ras GTPase, RNA binding, response to organic substance, cytokine-mediated signaling pathway, kinase regulatory activity, protein binding, positive regulation of neurogenesis, and alcohol biosynthesis process (Table 2). Detail changes in the gene sets regulating Ras GTPase, RNA binding, cytokine-mediated signaling pathway, protein binding, and alcohol biosynthesis process are illustrated (Figure 3A–3E). The respective gene components in these gene sets are included (Supplementary Tables 6–14). ETOP treatment also led to downregulations of gene sets regulating clathrin-coated vesicle, calmodulin binding, microtubule-based movement, and coated vesicle (Table 2; Figure 3F–3I; Supplementary Tables 15–18).

**Table 2:** Alteration of gene sets and pathways in ETOP-DEGs^a^

Gene sets	Set size^d^	*p*-value
GO:0032318 regulation of Ras GTPase activity^b^	11	0.0313
GO:0003723 RNA binding^b^	59	0.0391
GO:0010033 response to organic substance^b^	125	0.0399
GO:0019221 cytokine-mediated signaling pathway^b^	20	0.0423
GO:0019207 kinase regulator activity^b^	12	0.0445
GO:0071310 cellular response to organic substance^b^	88	0.0499
GO:0005515 protein binding^b^	491	0.0503
GO:0050769 positive regulation of neurogenesis^b^	12	0.0504
GO:0046165 alcohol biosynthetic process^b^	12	0.0506
R-HSA-72613: Eukaryotic Translation Initiation^b^	20	0.0202
R-HSA-72737: Cap-dependent Translation Initiation^b^	20	0.0202
R-HSA-72702: Ribosomal scanning and start codon recognition^b^	13	0.0202
R-HSA-72706: GTP hydrolysis and joining of the 60S ribosomal subunit^b^	19	0.0202
R-HSA-72649: Translation initiation complex formation^b^	13	0.0202
R-HSA-72662: Activation of the mRNA upon binding of the cap-binding complex and eIFs, and subsequent binding to 43S^b^	13	0.0202
R-HSA-72766: Translation^b^	23	0.0202
R-HSA-156827: L13a-mediated translational silencing of Ceruloplasmin expression^b^	18	0.0362
R-HSA-72695: Formation of the ternary complex, and subsequently, the 43S complex^b^	11	0.0449
GO:0030136 clathrin-coated vesicle^c^	19	0.0430
GO:0005516 calmodulin binding^c^	10	0.0452
GO:0007018 microtubule-based movement^c^	14	0.0471
GO:0030135 coated vesicle^c^	23	0.0495

^a^enrichment in gene sets and pathways was performed using the GAGE and Reactome packages in R

^b^the indicated gene sets were upregulated

^c^the indicated gene sets were downregulated

^d^number of DEGs that are enriched in the individual gene sets

GO gene sets were produced using the GAGE package

R-HSA gene sets were resulted using the Reactome package.

**Figure 4:**
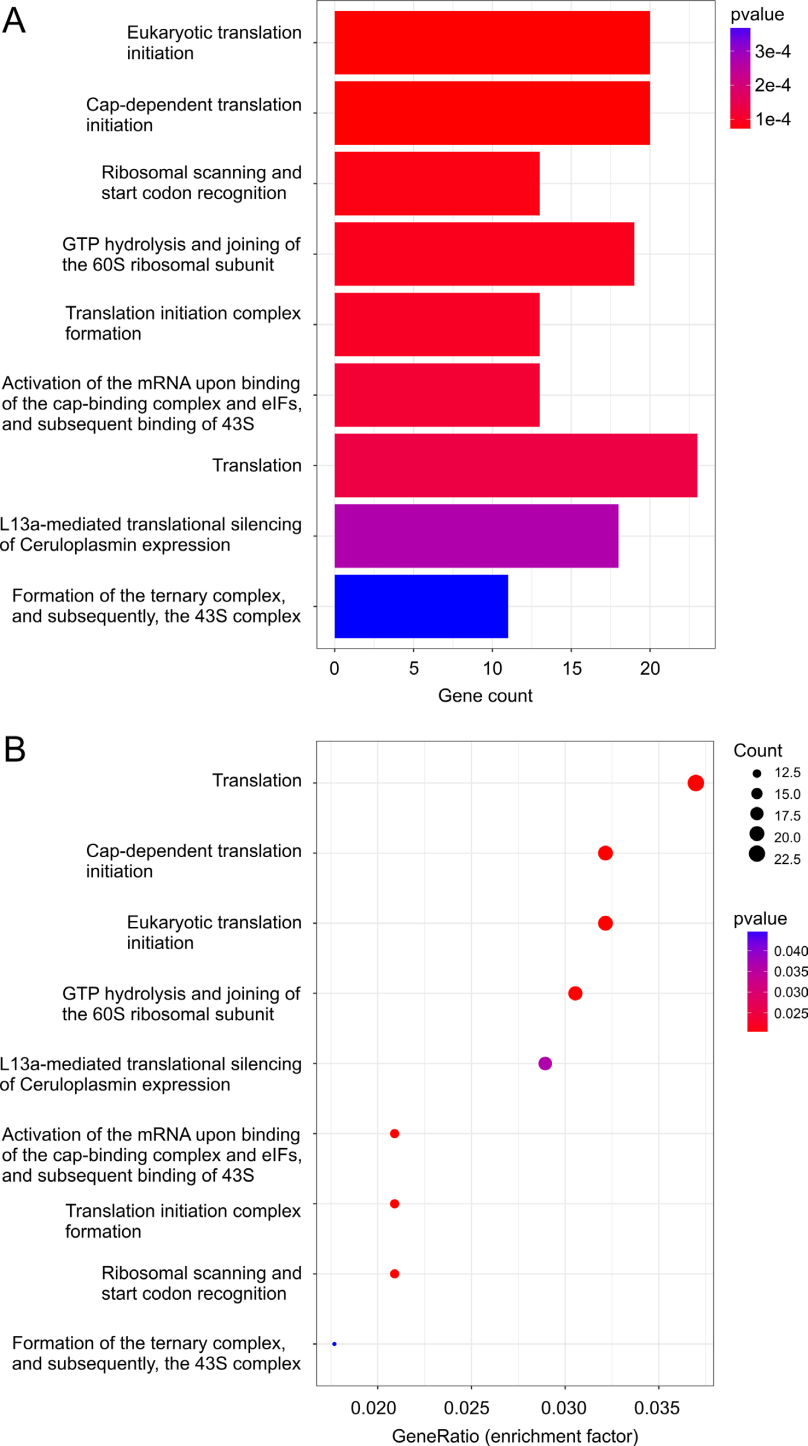
ETOP-induced DEGs are enriched in pathways regulating translation Pathway enrichment on ETOP-induced DEGs (*p* < 0.05; Supplementary Table 4) was carried out using the Reactome package in R [57]. The enriched pathway (**A**) and the enrichment factor (GeneRatio) (**B**) are graphed.

**Figure 5:**
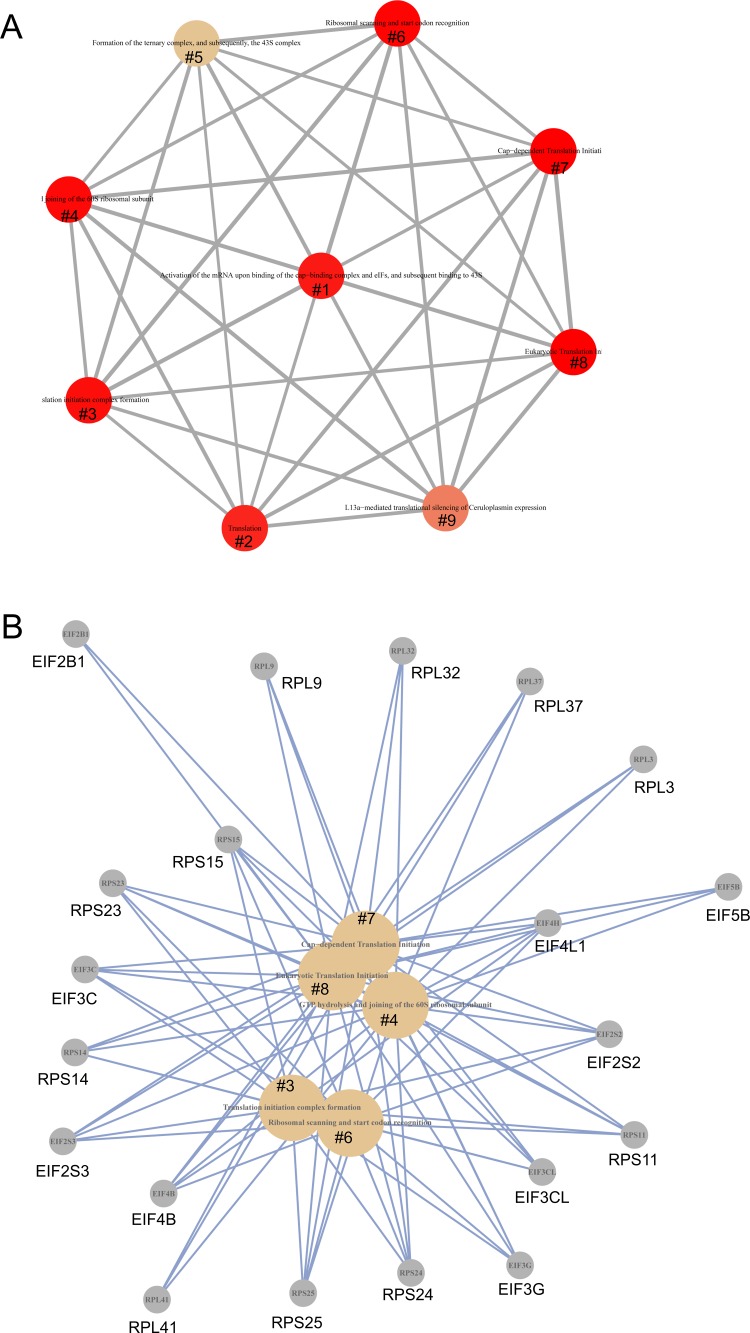
The network connection of the enriched pathways in protein translation Pathway enrichment analysis was performed using Network map of the Reactome package in R [57] on DEGs (*p* < 0.05). (**A**) Network of the enriched pathways. (**B**) Individual enriched pathways and their contributing DEGs. #1: Activation of the mRNA upon binding of the cap-binding complex and eIFs, and subsequent binding to 43S; #2: Translation; #3: Translation initiation complex formation; #4: GTP hydrolysis and joining of the 60S ribosomal subunit; #5: Formation of the ternary complex, and subsequently, the 43S complex; #6: Ribosomal scanning and start codon recognition; #7: Cap-dependent Translation Initiation; #8: Eukaryotic Translation Initiation; and #9: L13a-mediated translational silencing of Ceruloplasmin expression.

In comparison to vehicle treatment, analysis using Reactome revealed alterations in multiple aspects of translation in ETOP-treated MCF7 cells (Table 2; Supplementary Table 19). Alterations in these pathways and their enrichment factors are documented (Figure 4). The network nature of individual genes contributing to these enriched pathways is also demonstrated (Figure 5). Regulation of translation in DDR has been reported through selectively excluding and recruiting mRNA species to polysomes [11, 12]. Our research suggests that control of translation in DDR could also be occurred at the transcription level. Our observations are consistent with a recent RNA-seq analysis reporting alteration in translation in ETOP-treated mouse embryonic fibroblasts (MEFs) [58]. Taken together, the above observations revealed alterations in multiple pathways in MCF7 cells following ETOP treatment.

### Novel factors detected in ETOP-treated MCF7 cells

**Figure 6:**
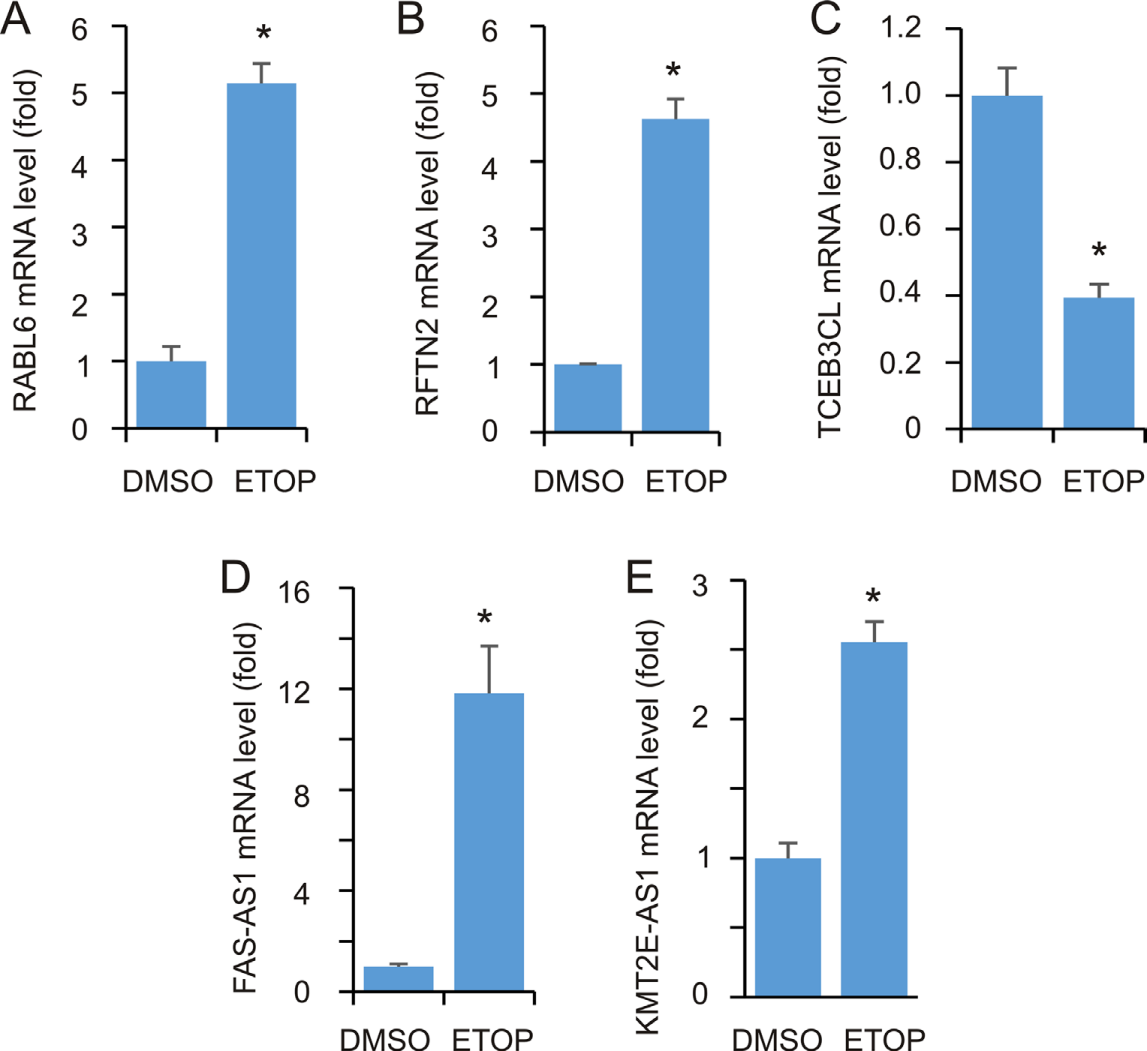
Real-time PCR analysis of DEGs MCF7 cells were treated with either vehicle or ETOP for 2 hours, followed by real-time PCR analysis for the indicated genes. Actin was used as a control; the expression of individual transcripts were normalized to actin, and expressed as fold changes to the control treatment. Experiments were repeated three times; ^*^: *p* < 0.05 in comparison to the control treatments (2-tailed Student’s *t-*test).

To further examine these DEGs, we have selected a few genes to confirm their alterations. These candidates were selected based on the following criteria. 1) Their involvement in DDR is largely unknown. 2) They are within different groups of DEGs. RABL6 is a DEG defined under FDR ≤ 0.001 (Supplementary Table 1) and RFTN2 upregulation appears in the DEG group of *p* < 0.05 (log2 ratio of ETOP/vehicle = 2.68, *p* = 0.052); the outcome would thus support our relax conditions used in the above analyses. 3) They potentially function in different processes (see later for details). Using real-time PCR, we demonstrated upregulations of RABL6 and RFTN2 (Figure 6A, 6B) and downregulation of TCEB3CL (Figure 6C).

We noticed the upregulation of several antisense (AS) RNA species in ETOP-treated cells (Supplementary Table 1). Since non-coding RNAs (ncRNAs) play critical roles in DDR regulation [32], we selected two AS species, one in the top (FAS-AS1) and another in the bottom (KMT2E-AS1) half of Supplementary Table 1, to examine their upregulation. As expected, real time PCR detected a robust FAS-AS1 upregulation (Figure 6D) and a significant KMT2E-AS1 increase (Figure 6E) following ETOP treatment.

**Figure 7:**
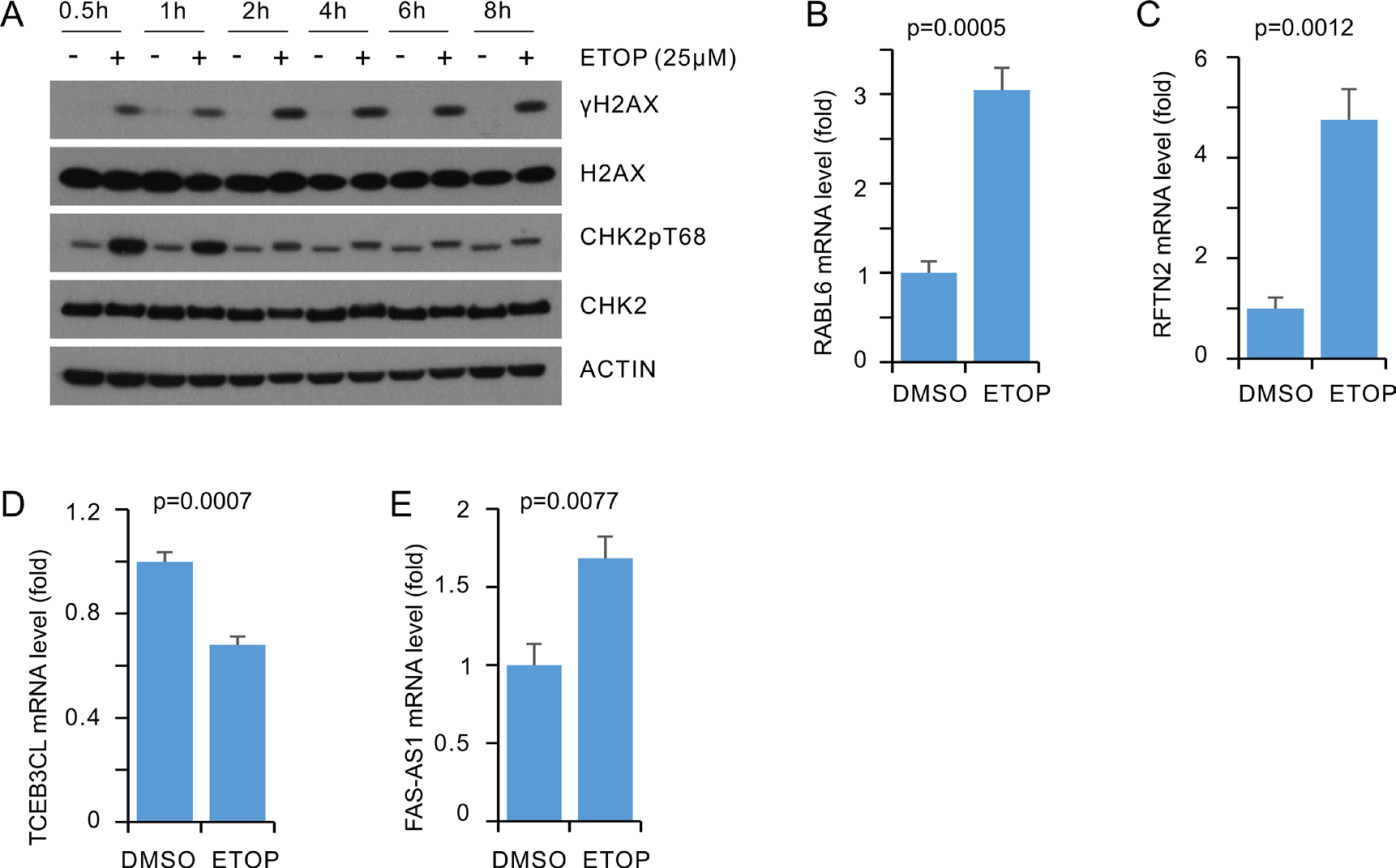
Real-time PCR analysis of DEGs in ETOP treated T47D cells T47D cells were treated with either vehicle or ETOP for 2 hours, followed by real-time PCR analysis for the indicated genes. Actin was used as a control; the expression of individual transcripts were normalized to actin, and expressed as fold changes to the control treatment. Experiments were repeated three times; ^*^: *p* < 0.05 in comparison to the control treatments (2-tailed Student’s *t-*test).

To examine whether the above alterations in MCF7 cells treated with ETOP is cell line specific, we have performed similar experiments using T47D breast cancer cells. First, ETOP at 25μM induced a plateau level of γH2AX, indicative of a peak level of DSBs, along with the induction of CHK2 T68 phosphorylation following 2 hour treatment in T47D cells (Figure 7A). ETOP treatment significantly upregulated RABL6, RFTN2, and FAS-AS1 (Figure 7B, 7C, 7E) but not KMT2E-AS1 (data not shown), and significantly downregulated TCEB3CL (Figure 7D) in T47D cells.

RAS oncogene family-like 6 (RABL6, RBEL1, pp8875, and C9orf86) is a member of the Ras family of small GTPase (https://www.ncbi.nlm.nih.gov/gene/55684) with a potential oncogenic role in part via inactivating the Rb1 tumor suppressor [59–61]. Its involvement in DDR has yet to be demonstrated.

RFTN2 (raftlin family member 2) was upregulated in ETOP-treated MCF7 cells (Figure 6B) and T47D cells (Figure 7C). There is currently lack of evidence suggesting its physiological functions.

The physiological roles, including DDR, of TCEB3CL (transcription elongation factor B polypeptide 3C-like) remain unknown.

While the biology of KMT2E-AS1 has not been reported, FAS-AS1 has been studied and named as Saf [62]. FAS-AS1 was demonstrated to protect T-lymphocytes and erythroblasts from FAS-induced apoptosis [62, 63], and has also been suggested to enhance FAS-mediated apoptosis in B-cell lympma by inhibiting the production of soluble FAS (sFAS) that prevents FAS-ligand from binding FAS [64]. More relevantly, upregulation of FAS-AS1 was reported following IR-induced DSBs [65]. Collectively, we provide the first evidence for a robust upregulation of FAS-AS1 following ETOP treatment, which may play a role in modulating ETOP-induced apoptosis in MCF7 cells.

### Dynamic alterations of transcription in MCF7 cells treated with ETOP

**Figure 8:**
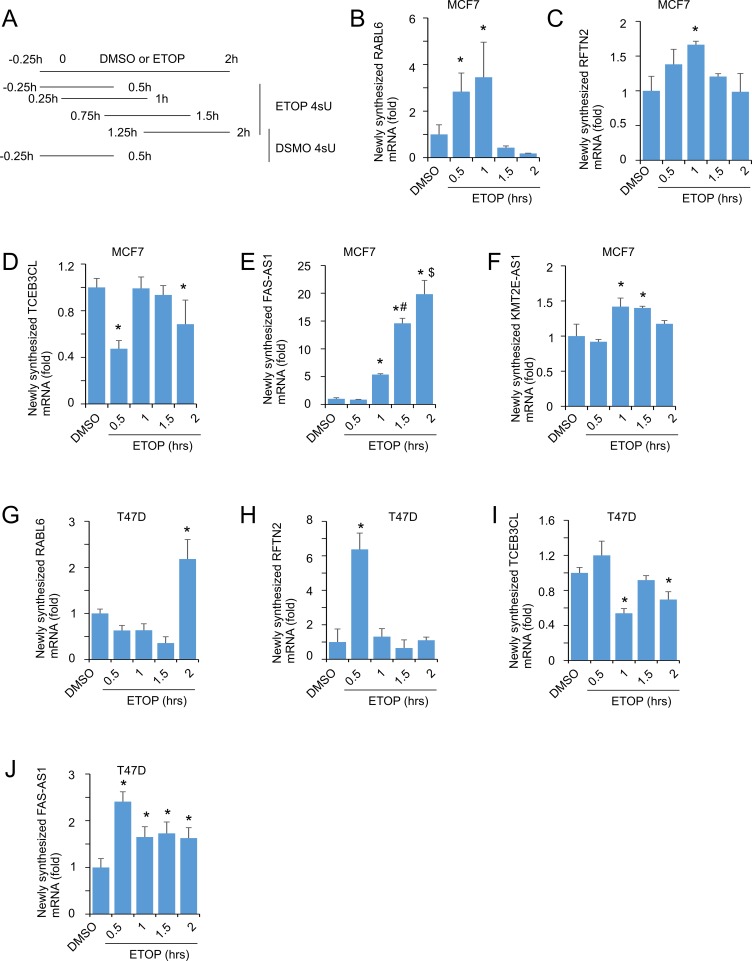
Alterations in RNA synthesis during DDR (**A**) Outline of a short term of metabolic labeling. DMSO and ETOP treated cells were labeled with 4sU at 250μM for 45 minutes as indicated. (**B**–**J**) Pre-existing RNA and newly synthesized (4sU-labelled) RNA was isolated and analyzed for the indicated transcripts. The human large 60S ribosomal protein L110E (H36B4) was used an internal control. The average levels of newly synthesized RNA in two periods of DMSO treatments (A) were used to compare to the rates of RNA synthesis for the indicated genes under ETOP treatment. All real time PCR reactions have normalized to H36B4. Experiments were repeated three times; means ± SD are graphed. ^*^p < 0.05 in comparison to DMSO; ^#^*p* < 0.05 compared to ETOP 1 h; ^$^*p* < 0.05 in compared to ETOP 1.5 h. Statistical analyses were performed using Student’s *t-*test (2-tailed).

RNA levels are affected by the rates of RNA synthesis and decay. To further determine whether the observed upregulation and downregulation of RNAs in ETOP-initiated DDR were attributable to RNA synthesis, we examined the rate of transcription of RABL6, RFTN2, TCEB3CL, FAS-AS1, and KMT2E-AS1 using the well-established 4-thiouridine (4sU)-based metabolic labeling system [66, 67]. The advantage of this system includes that 4sU does not affect RNA synthesis even after > 24 hour exposure at high doses [66]. Following published conditions [66, 68, 69], we performed a short duration (45 min) labelling covering 0.5 h, 15 – 60 min, 45 min – 90 min, and 75 min – 120 min of ETOP treatment (Figure 8A), and were able to show a significant enhancement in the synthesis of RABL6, RFTN2, FAS-AS1, and KMT2E-AS1 (Figure 8B, 8C, 8E, 8F), while the rate of TCEB3CL RNA synthesis was reduced in certain periods during the duration of ETOP treatment in MCF7 cells (Figure 8D). Dynamic alterations in the transcription of these target genes except KMT2E-AS1 were also demonstrated in T47D cells (Figure 8G–8J). These observations are in accordance with their alterations in the steady levels determined by RNA sequencing and real-time PCR analysis (Supplemntary Table 1 and Table 1; Figures 6 and 7). Collectively, these observations support the possibility that alterations in RNA levels during DDR are in part attributable to changes in gene transcription.

## DISCUSSION

An intimate relationship between RNA biology and DDR has been suspected for many decades. Insights on this concept have recently been emerged [19, 31–33]. An interesting aspect is the observed mutual impacts between transcription and DDR. The requirement of DSB, at least for the estrogen receptor target pS2 promoter, in transcription and AIRE-mediated transcription were intriguing [36, 37]. Recent development reveals a cross utilization of DNA damage repairing proteins as transcription factors and vice versa [70]. Despite these advances, our knowledge on the DDR-coupled transcription remains limited. DSBs initiate a local inhibition on transcription, a process that is regulated in part through BMI1-mediated histone ubiquitination [71]. However, whether DSBs and other DNA lesion types result in a global repression of transcription remains debating. We provide a unique analysis on DSBs induced by ETOP, and observe a high correlation coefficient 0.953 in more than 12,000 pairs of transcripts between vehicle-treated cells and ETOP-treated cells (Figure 1B). Thus, our and other’s research [28, 72] does not support a global transcription suppression by DNA damage. While global transcription inhibition can be envisaged under massive DNA damage, maintenance of an active global transcription under situations in which DNA damage can be repaired is likely critical for cells to manage the complex requirements: DDR execution and exit. This concept is supported by DDR regulating stem cell properties [73], and DNA damage modulating gene expression in other settings [74, 75].

Nonetheless, DNA damages at least ETOP-initiated DSBs modulate the expression of a set of genes (Supplementary Table 1, Table 1, Supplementary Tables 2–5). These genes include RAD52 upregulation (Supplementary Table 4), which is in accordance with the reported RAD52 increase in HepG2 cells following ETOP treatment [76]. The pathways affected by ETOP-induced differentially expressed genes are enriched in multiple processes and pathways that do not apparently relate to DNA lesion repair or checkpoint activation (Figures 2–5; Supplementary Tables 6–19). For example, the translation process is enhanced in ETOP-treated MCF7 cells (Table 2); RNA-seq analysis of MEFs treated with ETOP has also reported alteration in translation [58]. The biology of changes in translation in cells treated with ETOP is likely complex; future research will need to address this issue.

It remains largely unclear as to why the expression of selective genes is downregulated except for the reason to avoid complication with DNA damage repair. In contrast, evidence supports the concept that gene upregulation is to ensure the expression of DNA repair genes, a process in which p53 plays a role [33, 77]. However, IR also results in gene upregulation independent of p53 [30]. Collectively, our research together with the published evidence supports a much broad alteration in gene expression and a broad contribution of these alterations in the facilitations of cell fate selection under DNA damage. While these changes in RNA abundance can be attributable to alterations in the rates of RNA decay, our study using a limited number of genes (Figure 8) revealed a contribution of changes in RNA synthesis to the steady level of RNA during DDR. However, it should be stressed that our research does not exclude an impact RNA decay on RNA abundance in cells undergoing DDR.

The contributions of RNA to DDR is not limited to protein-coding mRNA. Noncoding RNAs (ncRNAs) are emerging as a critical regulator of DDR [31, 32], a development that fits well with the current knowledge that while approximately 70% of human genome is transcribed into RNAs, no more than 2% of the genome is for protein coding mRNAs [78]. Long noncoding (lncRNA) LINP1 enhances DSB repair through non-homologous end joining (NHEJ) [79]. While p53 inhibits LINP1 [79], p53 induces the production of lncRNA PANDA from the CDKN1A promoter in response to doxorubicin-induced DSB, and PANDA reduces the expression of pro-apoptotic factors [80]. Similar to the action of doxorubicin in poisoning topoisomerase II, we observed that ETOP also induces a set of ncRNAs, including FAS-AS1, KMT2E-AS1 (Supplementary Table 1; Figure 6D, 6E), SPTY2D1-AS1, and PSMD6-AS2 (Supplementary Table 1). FAS-AS1 regulates FAS-induced apoptosis [62–64], and is induced by IR through the action of ATM [65], a study that validates our work here. ETOP treatment also caused a downregulation of ncRNA Speedy hom E8 pseudogene (Table 1). While the physiological roles of these ncRNAs are unknown, it will be interesting to investigate their contributions to DDR. Since our studies are based on p53 wild type MCF7 cells, the contributions of p53 to the alterations of these genes could be studied in future.

In addition to our study using MCF7 cells, RNA-seq has been used to analyze ETOP-treated HEK293 [37], MEFs [58], and fetal liver-derived hematopoietic stem cells (FL-HSC) [81] for different purposes. While ETOP treatment activates the MAPK, WNT, JAK-STAT, SHH, and NOTCH pathways in FL-HSC [81], ETOP exposure alters the translation process [58] which is in line with our study. Nonetheless, our study represents a pioneer effort to directly address the global RNA expression in ETOP-induced DNA damage using the state-of-the-art RNA sequencing technology. However, our study should be cautiously interpreted. While ETOP induces DSBs, whether our observations here apply to cells undergoing DSBs induced by other genotoxic treatments should be investigated in the future.

While the current study focuses on ETOP-induced DDR, some alterations may also apply to other types of DDR. In supporting this possibility, we have detected the same changes in a limited number of genes in MCF7 cell treated with either ETOP or hydroxurea (HU, our unpublished observations). ETOP induces DDR via the production of DSBs and HU causes DDR via the induction of single strand DNA lesions [82, 83]. This commonality, nonetheless, is expected as DSBs and single strand DNA lesions co-occur in different DDR settings. However, unique pathways and DEGs are certainly expected in different types of DDR and in different types of cells. This area certainly deserves further investigations.

## MATERIALS AND METHODS

### Cell culture and DNA damage induction

MCF7 and T47D cells were purchased from American Type Culture Collection (ATCC; Manassas, VA), and cultured in DMEM supplemented with 10% Foetal Bovine Serum (FBS; Sigma Aldrich; Oakville, ON) and 1% Penicillin-Streptomycin (Life Technologies; Burlington, ON). Etoposide (ETOP) was obtained from Sigma. For induction of DSBs, cells were treated with either ETOP at 25 μM or vehicle (DMSO, 1:1000 dilution) for 2 hours.

### Western blot analysis

Western blot analysis was performed according to our established protocol [39, 54, 83–85]. Briefly, 50 µg of total cell lysate protein was separated on a SDS-PAGE gel and transferred onto Amersham Hybond ECL nitrocellulose membranes (Amersham, Baie d’Urfe, QC). Blots were treated with 5% skim milk and incubated at 4°C overnight with the following antibodies: anti-H2AX (1:1000, Santa Cruz), anti-γH2AX (1:1000, Upstate), anti-phosph-CHK2 (T68) (1:500, Cell Signaling), and anti-CHK2 (1:1000, Cell Signaling). The blots were then incubated with the specific HRP-conjugated secondary antibodies at room temperature for one hour, followed by developing signals using an ECL Western Blotting Kit (Amersham, Baie d’Urfe, QC).

### Real-time PCR analysis

Total RNA was isolated using TRIzol (Life Technologies, Burlington, ON) following the manufacturers’ instructions. Reverse transcription and qRT-PCR was carried out as previously described [86, 87]. Briefly, 2 µg of RNA was converted to cDNA, followed by qRT-PCR, where 1µL of cDNA was used in each reaction. Real time PCR primers used were presented in Supplementary Table 20.

### RNA sequencing analysis

RNA was extracted using mRNeasy Mini Kit (Qiagen, No. 217004) following the manufacturer’s instructions. RNA-seq libraries were prepared using TruSeq Ribo Profile Mammalian Kit (Illumina, RPHMR12126) as per manufacturer’s instructions, and sequenced by BGI using the HiSeq 4000 system. RNA sequences were quantified by BGI (http://www.genomics.cn/en/navigation/show_navigation?nid = 2657).

The number of raw reads obtained was 18,222,960 and 22,080,922 for vehicle and ETOP treated MCF7 cells, respectively. From raw reads, we removed the adaptor reads and low quality reads which were defined as reads containing more than 10% of unknown bases or reads consisting of > 50% of low quality reads. The clean reads obtained for the above two samples were respectively 99.97% and 99.96% of the individual raw reads, and were used to map to the HISAT reference genome [88] using the Bowtie2 alignment program [89]. Transcript quantification was performed using the RSEM program [90], and expressed as fragments per kilobase of exon per million fragments mapped (FPKM). FPKM was calculated based on the formula: FPKM = 10^6^C/(NL/10^3^), where C is the number of fragments aligned to a specific gene; N is the total number of fragment aligned to a gene; and L is the combined exon length (base number) of a gene. Therefore, FRKM is a normalized unit of transcript abundance, and can be used to compare gene expression among different genes. Clean reads have been deposited to NIH Sequence Read Archive (SRA) (access number: SRP104001).

### Screen for differentially expressed genes (DEGs)

DEGs in the pair of vehicle vs ETOP was screened using the Poisson Distribution Method based on a strict algorithm that was developed by BGI (http://www.genomics.cn/en/navigation/show_navigation?nid = 2657).

### Gene oncology (GO) enrichment analysis

This analysis was performed to analyze enrichment in all three GO ontologies (molecular function, cellular component and biological process) for DEGs derived from the comparison of vehicle vs ETOP. We first mapped DEGs to the GO terms as defined in the database (http://www.geneontology.org/), calculated gene numbers, and determined a significant enrichment using hypergeometric test. Significance (*p* < 0.05) was determined using Bonferroni Correction.

### Gene set and pathway enrichment analysis

The GAGE [56] and Reactome [57] packages in R were used to analyze DEGs for gene set and pathway enrichment within the go.set.hs databases [56].

### Analysis of RNA synthesis using metabolic labelling

MCF7 and T47D cells (10^6^) were labeling with 4-thiouridine (4sU) at 250 μM for 45 minutes at the treatment conditions defined in Figure 8A. The dose and duration used was based on publications reporting that this condition produces a sufficient level of labeling and without noticeable interference with RNA synthesis in mammalian cells [66, 68, 69]. RNA was isolated using miRNeasy MinElute; 4sU-labeled (newly synthesized) RNA in a total of 20 μg RNA was biotinylated using the EZ-Link Biotin-HPDP kit (Thermo Fisher Scientific) according the manufacturer’s protocol. Briefly, RNA was heated at 65°C and cooled on ice, followed by incubation with EZ-link Biotin HPDP (2 mg/ml dissolved in dimethylformamide/DMF) at room temperature for 2 hours in a labeling buffer (10mM Tris pH 7.4, 1 mM EDTA). Unbound Biotin-HPDP was removed by chloroform/isoamylalcohol (24:1) extraction. RNA was precipitated using isopropanol and washed with 75% ethanol. GlycoBlue Coprecipitant (Life Tech, AM9516), 15 μg was used as an RNA indicator. RNA was heated at 65°C. Biotinylated RNA was captured using conjugated MyOne Streptavidin C1 Beads (20 μl) (Invitrogen), which was prepared according to the manufacturer’s instruction, at room temperature for 90 minutes. The streptavidin C1 beads were then washed using a buffer (10 mM TrisCl, pH7.5; 1 mM EDTA; 1M NaCl) at 65°C for 1–2 minutes three times. The unlabeled RNA was collected by pooling supernatants and three washes. The beads were further washed at room temperature twice. The biotin-labelled RNAs were eluded using 100 mM dithiothreitol (DTT) at room temperature twice. The unlabeled RNAs and biotin-labelled RNAs were purified using RNeasy MiniElute Kits (Qiagen). RNAs were then used for RT and qPCR experiments.

### Statistical analysis

Difference in gene expression screened by the Poisson Distribution Method was determined using the Bonferroni method. A *p-*value < 0.05 was considered statistically significant. False discovery rate (FDR) [91] was calculated to correct both type I and type II errors. Student’s *t-*test was used to analyze real time PCR data with *p* < 0.05 being considered statistically significant.

## SUPPLEMENTARY MATERIALS TABLES






















