# Supplementary material for: Etoposide-induced DNA damage affects multiple cellular pathways in addition to DNA damage response
Source: Oncotarget. 2018 Feb 16;9(35):24122–39. doi: 10.18632/oncotarget.24517 (PMC5963631; doi:10.18632/oncotarget.24517)
Supplement: Supplementary file 1 [file oncotarget-09-24122-s001.pdf]

Etoposide-induced DNA damage affects multiple cellular pathways in addition to DNA damage response

SUPPLEMENTARY MATERIALS

Supplementary Table 1: Up-regulated genes in ETOP-treated MCF7 cells[1]. See_supplementary_Table 1

Supplementary Table 2: ETOP-upregulated genes at FDR < 0.05. See_supplementary_Table 2

Supplementary Table 3: ETOP-downregulated genes at FDR < 0.05. See_supplementary_Table 3

Supplementary Table 4: ETOP-upregulated genes at $p < 0.05$. See_supplementary_Table 4

Supplementary Table 5: ETOP-downregulated genes at $p < 0.05$. See_supplementary_Table 5

Supplementary Table 6: Details of the upregulated gene set of GO:0032318 regulation of Ras GTPase activity

Gene	sym	Crtllog2	ETOPlog2	D-C
85360	SYDE1	-6.64385619	4.046141782	10.68999797
9815	GIT2	-6.64385619	3.558267634	10.20212382
2041	EPHA1	-6.64385619	3.465974465	10.10983066
25780	RASGRP3	-6.64385619	2.72900887	9.37286506
5048	PAFAH1B1	-6.64385619	2.722466024	9.366322214
3268	AGFG2	-6.64385619	2.09423607	8.73809226
11153	FICD	1.459431619	4.662775172	3.203343553
10507	SEMA4D	2.731183242	4.44625623	1.715072988
64786	TBC1D15	3.160274831	4.62935662	1.469081789
9910	RABGAP1L	4.651912745	5.875042803	1.223130058
121512	FGD4	3.412781525	2.003602237	-1.409179288

Ctrl: control or vehicle treatment.

Supplementary Table 7: Details of the upregulated gene set of GO:0003723 RNA binding. See_supplementary_Table 7

Supplementary Table 8: Individual genes in the upregulated gene set of GO:0010033 response to organic substance. See_supplementary_Table 8

Supplementary Table 9: Individual genes in the upregulated gene set of GO:0019221 cytokine-mediated signaling pathway

Gene	sym	Crtllog2	ETOPlog2	D-C
3563	IL3RA	-6.64386	5.088311	11.73217
3665	IRF7	-6.64386	4.605257	11.24911
10379	IRF9	-6.64386	4.468583	11.11244
5970	RELA	-6.64386	3.968091	10.61195
59307	SIGIRR	-6.64386	3.65764	10.3015
5048	PAFAH1B1	-6.64386	2.722466	9.366322
816	CAMK2B	-6.64386	2.392317	9.036174
7187	TRAF3	-6.64386	2.321928	8.965784
5618	PRLR	-6.64386	0.799087	7.442943
3654	IRAK1	0.85599	3.937344	3.081355
3595	IL12RB2	0.443607	3.381283	2.937677
81858	SHARPIN	2.424922	4.785551	2.360628
6778	STAT6	3.258519	5.385431	2.126912
29965	C16orf5	2.849999	4.898208	2.048209
50615	IL21R	2.498251	3.923149	1.424898
5788	PTPRC	4.211012	5.623808	1.412796
3091	HIF1A	4.642124	5.753551	1.111427
3570	IL6R	6.488644	7.532161	1.043518
2209	FCGR1A	3.770829	-6.64386	-10.4147
5469	MED1	3.381283	1.333424	-2.04786

Supplementary Table 10: Individual genes in the upregulated gene set of GO:0019207 kinase regulator activity

Gene	sym	Crtllog2	ETOPlog2	D-C
9479	MAPK8IP1	-6.64386	4.055716	10.69957
26289	AK5	-6.64386	3.635754	10.27961
92335	STRADA	-6.64386	3.564378	10.20823
10411	RAPGEF3	-6.64386	2.097611	8.741467
5295	PIK3R1	-6.64386	1.967169	8.611025
51738	GHRL	2.347666	5.731183	3.383518
8503	PIK3R3	-0.34008	2.664483	3.004558
94032	CAMK2N2	3.748461	6.382321	2.63386
1026	CDKN1A	2.094236	4.635754	2.541518
5611	DNAJC3	2.503349	4.367371	1.864022
9467	SH3BP5	4.960234	6.299941	1.339707
10614	HEXIM1	5.364923	4.177121	-1.1878

Supplementary Table 11: Individual genes in the upregulated gene set of GO:0071310 cellular response to organic substance. See_supplementary_Table 11

Supplementary Table 12: Individual genes in the upregulated gene set of GO:0005515 protein binding. See_supplementary_Table 12

Supplementary Table 13: Individual genes in the upregulated gene set of GO:0050769 positive regulation of neurogenesis

Gene	sym	Crtllog2	ETOPlog2	D-C
5970	RELA	-6.64386	3.968091	10.61195
650	BMP2	-6.64386	2.978196	9.622052
23013	SPEN	-6.64386	2.948601	9.592457
5048	PAFAH1B1	-6.64386	2.722466	9.366322
5080	PAX6	-6.64386	1.847997	8.491853
4131	MAP1B	-1.4344	1.454176	2.888579
1002	CDH4	0.495695	2.833902	2.338207
10507	SEMA4D	2.731183	4.446256	1.715073
3611	ILK	3.357552	4.917432	1.55988
396	ARHGDIA	3.314697	4.808385	1.493689
6770	STAR	4.122673	5.381975	1.259303
3091	HIF1A	4.642124	5.753551	1.111427

Supplementary Table 14: Individual genes in the upregulated gene set of GO:0046165 alcohol biosynthetic process

Gene	sym	Crtllog2	ETOPlog2	D-C
54361	WNT4	-6.64386	4.702658	11.34651
22937	SCAP	-6.64386	4.125155	10.76901
5745	PTH1R	-6.64386	3.375735	10.01959
1040	CDS1	-6.64386	3.181103	9.824959
650	BMP2	-6.64386	2.978196	9.622052
3269	HRH1	-6.64386	2.462052	9.105909
3422	IDI1	2.65764	5.537296	2.879656
51477	ISYNA1	3.431623	5.076388	1.644765
6770	STAR	4.122673	5.381975	1.259303
6309	SC5DL	2.967169	4.0917	1.124531
2247	FGF2	4.763943	2.604071	-2.15987
23541	SEC14L2	5.423578	4.113534	-1.31004

Supplementary Table 15: Individual genes in the downregulated gene set of GO:0030136 clathrin-coated vesicle

Gene	sym	Crtllog2	ETOPlog2	D-C
9026	HIP1R	-6.64386	2.689299	9.333155
10228	STX6	-6.64386	2.443607	9.087463
55361	PI4K2A	0.097611	3.564378	3.466767
26503	SLC17A5	2.698218	4.505891	1.807672
23208	SYT11	1.739848	3.528571	1.788723
3949	LDLR	3.848998	5.532005	1.683007
3092	HIP1	3.228049	4.357552	1.129503
6517	SLC2A4	4.862947	5.891905	1.028958
127833	SYT2	2.666757	3.674687	1.00793
2209	FCGR1A	3.770829	-6.64386	-10.4147
1173	AP2M1	3.537296	-6.64386	-10.1812
8120	AP3B2	2.845992	-6.64386	-9.48985
4643	MYO1E	2.613532	-6.64386	-9.25739
1889	ECE1	2.550901	-6.64386	-9.19476
2902	GRIN1	4.275007	0.070389	-4.20462
164	AP1G1	2.192194	-0.73697	-2.92916
8898	MTMR2	2.769772	-0.152	-2.92177
7851	MALL	4.494416	2.130931	-2.36348
5286	PIK3C2A	3.578939	2.301588	-1.27735

Supplementary Table 16: Individual genes in the downregulated gene set of GO:0005516 calmodulin binding

Gene	sym	Crtllog2	ETOPlog2	D-C
816	CAMK2B	-6.64386	2.392317	9.036174
4628	MYH10	2.538538	5.32589	2.787352
3784	KCNQ1	2.885574	4.44228	1.556706
4645	MYO5B	2.813525	-6.64386	-9.45738
4643	MYO1E	2.613532	-6.64386	-9.25739
5136	PDE1A	2.192194	-6.64386	-8.83605
3756	KCNH1	1.475085	-6.64386	-8.11894
2902	GRIN1	4.275007	0.070389	-4.20462
4638	MYLK	4.847496	2.217231	-2.63027
84033	OBSCN	2.185867	0.765535	-1.42033

Supplementary Table 17: Individual genes in the down-regulated gene set of GO:0007018 microtubule-based movement

Gene	sym	Crtllog2	ETOPlog2	D-C
5048	PAFAH1B1	-6.64386	2.722466	9.366322
4131	MAP1B	-1.4344	1.454176	2.888579
144132	DNHD1	-0.73697	1.83996	2.576925
3835	KIF22	2.06695	4.268285	2.201334
81027	TUBB1	4.36247	6.307611	1.945141
4134	MAP4	2.885574	4.382667	1.497093
3091	HIF1A	4.642124	5.753551	1.111427
10376	TUBA1B	5.12143	-6.64386	-11.7653
374654	KIF7	3.140779	-6.64386	-9.78463
64147	KIF9	3.125982	-6.64386	-9.76984
22820	COPG	2.851999	-6.64386	-9.49586
8120	AP3B2	2.845992	-6.64386	-9.48985
5870	RAB6A	3.725741	1.531069	-2.19467
998	CDC42	4.544114	2.643856	-1.90026

Supplementary Table 18: Individual genes in the downregulated gene set of GO:0030135 coated vesicle

Gene	sym	Crtllog2	ETOPlog2	D-C
22937	SCAP	-6.64386	4.125155	10.76901
9026	HIP1R	-6.64386	2.689299	9.333155
10228	STX6	-6.64386	2.443607	9.087463
55361	PI4K2A	0.097611	3.564378	3.466767
9276	COPB2	0.516015	3.566815	3.0508
26503	SLC17A5	2.698218	4.505891	1.807672
23208	SYT11	1.739848	3.528571	1.788723
3949	LDLR	3.848998	5.532005	1.683007
3092	HIP1	3.228049	4.357552	1.129503
6517	SLC2A4	4.862947	5.891905	1.028958
127833	SYT2	2.666757	3.674687	1.00793
2209	FCGR1A	3.770829	-6.64386	-10.4147
1173	AP2M1	3.537296	-6.64386	-10.1812
22820	COPG	2.851999	-6.64386	-9.49586
8120	AP3B2	2.845992	-6.64386	-9.48985
4643	MYO1E	2.613532	-6.64386	-9.25739
1889	ECE1	2.550901	-6.64386	-9.19476
2902	GRIN1	4.275007	0.070389	-4.20462
59349	KLHL12	4.953265	1.895303	-3.05796
164	AP1G1	2.192194	-0.73697	-2.92916
8898	MTMR2	2.769772	-0.152	-2.92177
7851	MALL	4.494416	2.130931	-2.36348
5286	PIK3C2A	3.578939	2.301588	-1.27735

Supplementary Table 19: ETOP-induced alterations in pathways determined using Reactome in R.
See_supplementary_Table 19

Supplementary Table 20: Real-time PCR primers

RABL6	GAGAGCGAGGGATCAGACAC
	TTTGCCCTCCTTACCTTCCT
RFTN2	TGGCTGAATTCGGATGGCTT
	GGCGGCTGGCTTTCTTATCT
TCEB3CL	CTCAGAAATCGCCTCCTGTC
	GAGAGTGCTTCTGGGTTTGC
FAS-AS1	TCAGAGGCTCCGGTACTCAA
	CAGAACCCGGCGCCTATTAT
KMT2E-AS1	GAGAAGGTTTGGCGCCCTAT
	GTTGATTCCGAGTCCCACGT
Actin	ACCGAGCGCGGCTACAG
	CTTAATGTCACGCACGATTTCC
