# Supplementary material for: Etoposide-induced DNA damage affects multiple cellular pathways in addition to DNA damage response
Source: Oncotarget. 2018 Feb 16;9(35):24122–39. doi: 10.18632/oncotarget.24517 (PMC5963631; doi:10.18632/oncotarget.24517)
Supplement: Supplementary file 2 [file oncotarget-09-24122-s002.docx]

**Table 1.** Up-regulated genes in ETOP-treated MCF7 cells^1^

| Gene | locus | Log2 Ratio | P value | FDR |
| --- | --- | --- | --- | --- |
| CCDC85B | 11q13.1 | 13.72 | 2.51E-12 | 7.73E-10 |
| B3GNT4 | 12q24.31 | 12.66 | 1.65E-07 | 2.39E-05 |
| PLGLB2 | 2p11.2 | 12.63 | 9.61E-14 | 4.09E-11 |
| C11orf31 | 11q12.1 | 12.63 | 4.30E-06 | 0.000402 |
| CCDC64B | 16p13.3 | 12.55 | 4.47E-08 | 6.97E-06 |
| FAS-AS1 | 10q23.31 | 12.47 | 1.17E-06 | 0.00014 |
| TSSK2 | 22q11.21 | 12.29 | 6.08E-07 | 7.80E-05 |
| SPDYE2 | 7q22.1 | 12.12 | 1.17E-06 | 0.000141 |
| MTUS2 | 13q12.3 | 12.01 | 4.82E-12 | 1.41E-09 |
| ACP2 | 11p12-p11 | 11.95 | 8.26E-06 | 0.000657 |
| PPM1H | 12q14.1-q14.2 | 11.78 | 9.98E-16 | 5.35E-13 |
| VPS37B | 12q24.31 | 11.48 | 4.30E-06 | 0.000405 |
| WNT4 | 1p36.12 | 11.35 | 1.65E-07 | 2.36E-05 |
| FAM222B | 17q11.2 | 10.81 | 8.26E-06 | 0.000644 |
| SCAP | 3p21.31 | 10.77 | 8.26E-06 | 0.000649 |
| AHDC1 | 1p36.11-p35.3 | 10.15 | 8.26E-06 | 0.000653 |
| SESN3 | 11q21 | 9.71 | 4.30E-06 | 0.000399 |
| SPEN | 1p36.21-p36.13 | 9.59 | 3.16E-07 | 4.38E-05 |
| WISP1 | 8q24.22 | 5.36 | 2.26E-12 | 7.33E-10 |
| JMY | 5q14.1 | 5.07 | 7.73E-19 | 5.30E-16 |
| TRERF1 | 6p21.1 | 4.79 | 5.21E-08 | 8.03E-06 |
| HCFC1R1 | 16p13.3 | 4.77 | 1.15E-16 | 6.75E-14 |
| NLRC4 | 2p22.3 | 4.47 | 2.14E-06 | 0.000229 |
| MYO15B | 17q25.1 | 4.41 | 3.96E-06 | 0.000381 |
| MED26 | 19p13.11 | 4.41 | 3.96E-06 | 0.000384 |
| ZNF286B | 17p11.2 | 4.36 | 6.23E-07 | 7.83E-05 |
| NNAT | 20q11.23 | 4.03 | 3.53E-09 | 6.40E-07 |
| C9orf131 | 9p13.3 | 3.99 | 3.41E-12 | 1.03E-09 |
| SGSM2 | 17p13.3 | 3.92 | 7.14E-08 | 1.06E-05 |
| XYLT2 | 17q21.33 | 3.85 | 1.23E-10 | 2.97E-08 |
| DDX11L2 | 2q14.1 | 3.83 | 6.23E-07 | 7.91E-05 |
| PCDH12 | 5q31.3 | 3.74 | 1.29E-12 | 4.55E-10 |
| NBPF8 | 1p11.2 | 3.72 | 2.22E-10 | 4.89E-08 |
| DARS2 | 1q25.1 | 3.68 | 1.39E-06 | 0.000162 |
| RDH13 | 19q13.42 | 3.58 | 1.23E-10 | 2.91E-08 |
| ZBTB44 | 11q24.3 | 3.53 | 5.47E-21 | 4.81E-18 |
| ZNF629 | 16p11.2 | 3.43 | 7.83E-10 | 1.56E-07 |
| PDZD2 | 5p13.3 | 3.36 | 2.66E-13 | 1.06E-10 |
| ZNF687 | 1q21.3 | 3.31 | 7.93E-11 | 1.99E-08 |
| RABL6 | 9q34.3 | 3.31 | 1.60E-18 | 1.04E-15 |
| RASSF2 | 20p13 | 3.30 | 1.36E-08 | 2.33E-06 |
| KRTAP5-2 | 11p15.5 | 3.20 | 2.40E-06 | 0.000249 |
| C12orf61 | 12q14.1 | 3.15 | 4.28E-11 | 1.12E-08 |
| ADAMTSL4 | 1q21.2 | 3.05 | 1.29E-05 | 0.000941 |
| GTF2H2C | 5q13.2 | 2.99 | 2.43E-12 | 7.67E-10 |
| ALDOA | 16p11.2 | 2.98 | 3.40E-10 | 7.34E-08 |
| DEAF1 | 11p15.5 | 2.93 | 1.97E-10 | 4.42E-08 |
| PHPT1 | 9q34.3 | 2.88 | 6.10E-09 | 1.07E-06 |

**Table 1.** Continued

| Gene | locus | Log2 Ratio | P value | FDR |
| --- | --- | --- | --- | --- |
| RASGRP1 | 15q14 | 2.88 | 3.54E-06 | 0.000349 |
| TLK1 | 2q31.1 | 2.81 | 6.60E-09 | 1.15E-06 |
| MTSS1 | 8q24.13 | 2.81 | 1.64E-10 | 3.81E-08 |
| HSD3BP4 | 1p12 | 2.79 | 4.22E-06 | 0.0004 |
| MYH10 | 17p13.1 | 2.79 | 1.53E-12 | 5.10E-10 |
| SNRK | 3p22.1 | 2.79 | 1.04E-05 | 0.000799 |
| LRIG1 | 3p14.1 | 2.64 | 4.70E-06 | 0.000426 |
| CLK3 | 15q24.1 | 2.53 | 3.28E-07 | 4.49E-05 |
| GJA9-MYCBP | 1p34.3 | 2.52 | 6.76E-06 | 0.00056 |
| LOC148709 | 1q32.1 | 2.50 | 4.70E-06 | 0.000423 |
| SLC24A1 | 15q22.31 | 2.50 | 2.33E-09 | 4.35E-07 |
| KMT2E-AS1 | 7q22.3 | 2.48 | 5.80E-06 | 0.000496 |
| ANGPT2 | 8p23.1 | 2.47 | 4.26E-11 | 1.14E-08 |
| PRSS12 | 4q26 | 2.46 | 5.95E-10 | 1.26E-07 |
| SPIRE2 | 16q24.3 | 2.43 | 7.94E-06 | 0.000636 |
| C9orf172 | 9q34.3 | 2.33 | 2.21E-11 | 6.04E-09 |
| FHOD1 | 16q22.1 | 2.29 | 1.35E-09 | 2.60E-07 |
| THUMPD3 | 3p25.3 | 2.25 | 1.80E-16 | 1.01E-13 |
| SPTY2D1-AS1 | 11p15.1 | 2.17 | 1.33E-38 | 4.10E-35 |
| PTK6 | 20q13.33 | 2.16 | 2.40E-08 | 3.95E-06 |
| RAB6B | 3q22.1 | 2.16 | 2.40E-08 | 4.00E-06 |
| ATXN7 | 3p14.1 | 2.12 | 1.58E-11 | 4.42E-09 |
| C14orf23 | 14q12 | 2.10 | 1.96E-06 | 0.000218 |
| UTP11L | 1p34.3 | 2.09 | 8.36E-21 | 6.86E-18 |
| LIPT1 | 2q11.2 | 2.09 | 1.96E-06 | 0.00022 |
| TMEM130 | 7q22.1 | 1.97 | 2.13E-06 | 0.00023 |
| TMEM115 | 3p21.31 | 1.96 | 5.36E-18 | 3.30E-15 |
| TUBB1 | 20q13.32 | 1.95 | 7.06E-08 | 1.06E-05 |
| ZHX3 | 20q12 | 1.91 | 4.04E-07 | 5.41E-05 |
| CPM | 12q15 | 1.87 | 4.93E-34 | 1.22E-30 |
| MPP5 | 14q23.3 | 1.83 | 3.68E-06 | 0.00036 |
| MAMDC4 | 9q34.3 | 1.82 | 2.38E-15 | 1.22E-12 |
| LDLR | 19p13.2 | 1.68 | 1.28E-05 | 0.000941 |
| LRPAP1 | 4p16.3 | 1.60 | 5.22E-24 | 7.15E-21 |
| RNASEH2C | 11q13.1 | 1.59 | 4.54E-11 | 1.17E-08 |
| CACNA2D2 | 3p21.31 | 1.58 | 4.64E-07 | 6.15E-05 |
| LOC100133091 | 7q11.23 | 1.54 | 8.10E-11 | 2.00E-08 |
| NCOA3 | 20q13.12 | 1.33 | 5.41E-06 | 0.000483 |
| NFAT5 | 16q22.1 | 1.25 | 5.67E-06 | 0.000495 |
| CARM1 | 19p13.2 | 1.25 | 1.49E-12 | 5.10E-10 |
| ACOT13 | 6p22.3 | 1.25 | 3.49E-13 | 1.34E-10 |
| RABGAP1L | 1q25.1 | 1.22 | 1.14E-05 | 0.00086 |
| NSUN5 | 7q11.23 | 1.22 | 1.38E-14 | 6.53E-12 |
| CENPP | 9q22.31 | 1.09 | 3.39E-06 | 0.000337 |
| TMEM184A | 7p22.3 | 1.06 | 1.20E-06 | 0.000142 |
| IL6R | 1q21.3 | 1.04 | 1.09E-05 | 0.000829 |
| PSMD6-AS2 | 3p14.1 | 1.01 | 5.84E-07 | 7.58E-05 |
| PPM1E | 17q22 | 1.00 | 7.81E-10 | 1.58E-07 |

1: The candidates were selected based on log2 ratio (ETOP/vehicle) ≥ 1 and FDR ≤ 0.001
